# Isolation and investigation of external reproduction tract mycoflora in healthy male cats

**DOI:** 10.1002/vms3.1351

**Published:** 2024-01-16

**Authors:** Massoud Talebkhan Garoussi, Aghil Sharifzadeh, Mahssa Khodabakhsh, Abdol Ali Malmasi

**Affiliations:** ^1^ Faculty of Veterinary Medicine Department of Theriogenology University of Tehran Tehran Iran; ^2^ Faculty of Veterinary Medicine Mycology Research Centre University of Tehran Tehran Iran; ^3^ Faculty of Veterinary Medicine Department of Internal Medicine University of Tehran Tehran Iran

**Keywords:** cat, external organs, genitalia, male, mycoflora

## Abstract

**Background:**

Various infectious agents can infect the male reproductive system.

**Objectives:**

The aims of this study were to provide current data on fungal and yeast flora of the external organs of reproductive system of male short hair cats including penis and prepuce.

**Methods:**

In total 28 samples were taken from external genital system of male stray cats using sterilised cotton swabs. Samples were taken based on the absence of any reproductive complications using physical examinations. The samples were transferred to sterilised Stuart transport media and were then incubated in the Sabouraud dextrose agar with chloramphenicol at 32°C for 48 h. The identification of fungi and yeasts was confirmed by germ tube formation test, CHROM agar, urease testing and Corn meal agar medium with Tween 80.

**Results:**

Fungal agents were isolated from 7 (25%) out of 28 male stray cats. Fungal isolates were obtained from the penis and prepuce of 4 (36%) and 7 (100%) male cats, respectively. The most commonly recovered species samples were *Candida krusei* (3.75%) and *Penicillum* spp. (6.86%) from the penis and prepuce of the cats, respectively. The external organs of male reproduction of cats were infected with 2–4 different fungal agents. Only 5 (45%) cats were infected with one fungus; however, in 6 (55%) cats, mixed fungal infections were isolated. Cats 3–4 years old had the highest fungal contamination in the prepuce region (4/7), while the penis at this age had the least contamination (1/4).

**Conclusions:**

It is concluded that the external reproduction organs of male cats could be infected by different fungal agents.

## Graphical abstract

Less data are available for description of fungal isolates of normal reproduction of male cats. This study provided current data on fungi and yeast flora of the external organs of reproduction system of male short hair stray cats included penis and prepuce. We observed the occurrence of fungal contamination mainly *Penicillium* and *Candida*. These microorganisms are likely to be either transient or resident due to predisposing factors such as diabetes and immunodeficiency in the male reproductive system of stray cats.

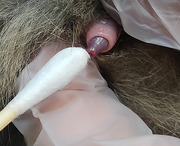

## 1 INTRODUCTION

Many different infectious agents can lead to male reproduction system diseases in animals. Some infectious agents have their primary target such as the genital tract. Therefore, it is important to detect continuous or occasional genital system microbial populations. It is important to understand their potential role(s) as an endogenous source of infection. Knowledge of the infectious agents living in the male animals’ genital systems environment is relevant to a better understanding of these pathological processes. Little data on the impact of infections on the reproduction system have been described in male cats in general. Mycoflora of different organs of the genital system in female animals with or without reproductive problems have been described as normal and pathologic fungal agents (Massoud Talebkhan Garoussi et al., 2007; Talebkhan Garoussi et al., 2008). Mammary glands could also be infected by pathologic fungal agents as normal fungal agents as normal flora (Massoud Talebkhan Garoussi et al., 2012). The first isolated fungus from a bovine uterus was reported in 1920 (Smith, 1920). Mycotic abortion caused by *Aspergillus*, *Candida*, *Zygomycetes* and yeasts has been observed in cows and buffalos (Ainsworth & Austwick, 1973). Endometritis and cervicitis due to fungal infection in mares has been described (Blue, 1983; Collins, 1964; Pugh et al., 1986). However, some mycotic agents can be pathologic in cows (Massoud Talebkhan Garoussi et al., 2007; Talebkhan Garoussi et al., 2008), dogs (Ismar et al., 2004) and horses (Chengappa et al., 1984).

Fungi such as *Candida albicans* (*C. albicans*) and *C. globrata* are found in the normal vaginal flora of canines and vary according to the stage of the oestrous cycle, which is known to constitute an endogenous source of infection (Cleff et al., 2001). In humans, the importance of fungi in reproduction is demonstrated by the prevalence of vulvovaginitis (mainly *Candida*) in women (Sobel & Chaim, 1996). It has been estimated that approximately 75% of women experience at least one episode of vulvovaginitis during their lifetime (Fidel et al., 2000).

Vaginal fungal infections of owned, stray and existing cats in dairy cattle farms were examined (Garoussi et al., 2016). *Penicillium* was the most commonly isolated fungal infection. However, stray cats (4%) and cats in dairy farms (3%) were also infected with *Aspergillus* (A.) and *C. Krusei*, respectively.

Research on infection of the reproductive system of male animals has received less attention. However, most of the studies regarding flora of the genital systems in animals have been in females including cows, horses, camels and dogs (Azarvandi et al., 2017a, 2017b; Cleff et al., 2005, 2001; Massoud Talebkhan Garoussi et al., 2007; Różański et al., 2013a, 2013b; Shokri et al., 2010; Talebkhan Garoussi et al., 2008). Several studies have been performed exploring disorders of the female genital system and nongenital systems in cats (Garoussi et al., 2016; Khosravi et al., 2008). Also, less data are available both for description of fungal isolates of normal reproduction and for management of common problems in different kinds of domestic feline populations.

Because of their potential role as an endogenous source of infection, identifying the microorganisms of the normal penile flora will contribute to our understanding of their role in inflammation and disorders of the genital system of male cats. Due to the fact that until now, there has not been a necessary investigation on the fungal contamination of the reproductive system of male cats, so this issue seems necessary, because domestic cats may also have the same type of contamination which is considered as a sanitation hazard for human. In the investigations conducted on other animals, fungi were isolated that were different from the fungi isolated from such animals (Talebkhan Garoussi et al., 2008; Garoussi et al., 2016; Shokri et al., 2010). On the other hand, most of the investigations were on female animals, not male animals. Therefore, the innovation of this study is to investigate the fungal contamination of the reproductive system of male cats. However, the purpose of this study was to provide current data on fungi and yeast flora of the external organs of the reproduction system of male short hair cats including penis and prepuce.

## 2 MATERIAL AND METHODS

### 2.1 Sampling

**FIGURE 1:**
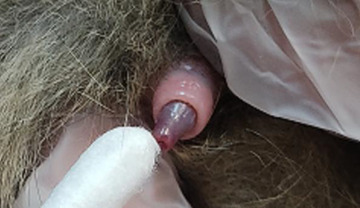
Male cat penis fungal sampling using sterilised cotton swabs for fungal cultures.

Samples were obtained from the external genital system of 28 short hair stray male cats. The animals were chosen based on no genital infections noted on physical examination. Samples were taken from the penis and prepuce using sterilised cotton swabs for fungal cultures (Figure 1). Three swabs were taken from each site. The samples were transferred to sterilised Stuart transport media (Difco Laboratories, USA).

### 2.2 Isolation and identification of fungi

**FIGURE 2:**
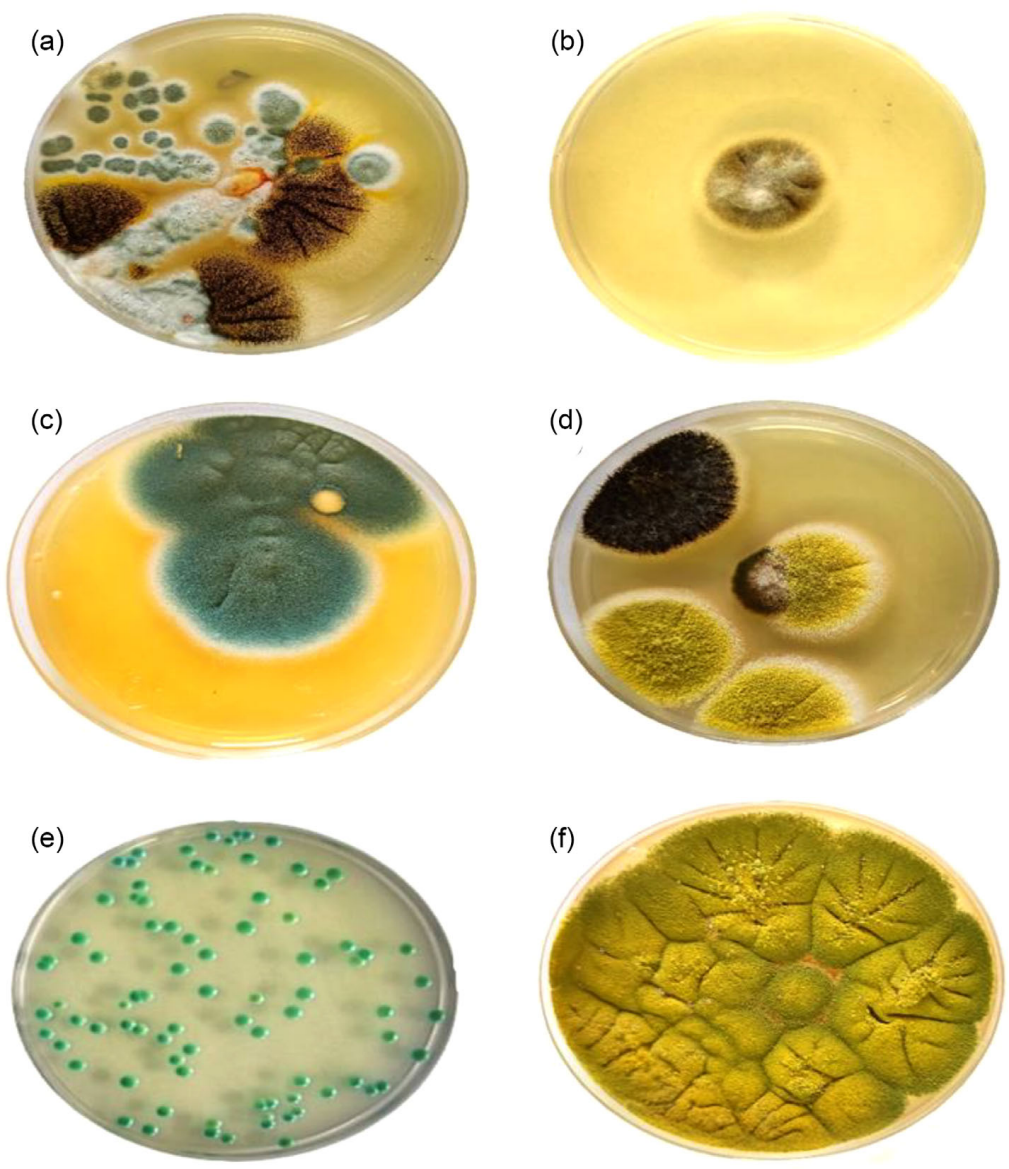
Macroscopic images of some fungal colonies isolated from the external reproductive tract of healthy male stray cats for identification. Colonies grown on Saburo dextrose agar included (A) *Aspergillus niger* (black colonies) and *Penicillium*. spp. (green colonies), (B) *Alternaria*. spp., (C) *Aspergillus fumigatus*, (D) *Aspergillus niger* (black colony) and *Aspergillus flavus* (light green colonies), (F) *Aspergillus flavus*, (E) light green colonies of *Candida albicans* on CHROM^TM^ Candida agar.

Cotton swabs were inoculated aerobically on to Sabouraud dextrose agar (Merck Co., Darmstadt, Germany). They were supplemented with antibiotic (chloramphenicol; 0.005%) and kept at 30°C for 7−10 days before being considered negative. Sterilised sealing film was used to cover each culture dish. Colonies were examined under a light microscope to determine the morphological structures of yeasts on slides mounted in Lactophenol cotton blue. Yeast colonies grown on Sabouraud dextrose agar were cultured linearly on CHROM agar^TM^ *Candida* (CHROMagar, France) and kept at 35°C for 48 h, and then according to the specific colour created in the culture medium, *C. albicans, C. krusei* and *C. tropicalis* were identified as light green, pink‐fuzzy and metallic blue, respectively (Figure 2A–F). In the case of colonies without a specific colour to distinguish species, a commercial RapID^TM^ Yeast Plus System kit (Remel, USA) according to the instruction of manufacture was used. This kit was designed based on biochemical tests such as sugar fermentation and assimilation, amino acids assimilation and urease activity. The morphology of any fungus was identified by examining a small aliquot of the colony in Lactophenol cotton blue staining under dry objectives (940) after carefully separating the fungal mass, with a fine straight dissecting needle, on to a slide (Madrid et al., 2012). Age of the cats was estimated and recorded (Tobias et al., 1998).

### 2.3 Sample size and statistical analysis

The minimum sample size required to estimate the prevalence of male external genital fungal contamination with a level of confidence of 95%, desired absolute precision of 10% and an expected prevalence of 90%, was found to be 24 male cats (Thrusfield, 2018).

Data were analysed using Genmod procedure including function link logit in the model. Differences at *p* < 0.05 were considered statistically significant.

## 3 RESULTS

In this study, fungi were isolated from the external genitalia of 7 (25%) of the 28 male stray cats. Fungal isolates were obtained from the penial and prepuce of 4 (36%) and 7 (100%) male cats, respectively (Table 1). Cats 3–4 years old had the highest incidence of fungal infection in the preputial region (4/7). There were no significant differences among the different organs and ages of the cats (*p* > 0.05).

**TABLE 1:** Distribution of mycoflora of external organs of reproduction system with different ages of male stray cats.

Organs	Isolates		Total
	+(%)	–(%)	
Penis (%)	4 (14)	24 (86)	28
Prepuce (%)	7 (25)	21 (75)	28
Total	11 (20)	45 (80)	56

Age (years)	Isolates		
Organs	+(%)	–(%)	Total
Penis (%)
≤2	1 (4)	12 (43)	13
3–4	1 (4)	9 (32)	10
≥5	2 (7)	3 (11)	5
Prepuce (%)			
≤2	1 (4)	12 (43)	13
3–4	4 (14)	6 (21)	10
≥5	2 (7)	3 (11)	5

It was shown that only four cats had infection in both organs. However, only three cats were contaminated in the prepuce region. The most commonly recovered species sampled *C. Krusei* (3.75%) and *Penicillum* spp. (6.86%) from the penis and prepuce of the cats, respectively (Table 2). Seven different isolates were recovered. Some cats were affected by four different fungi isolated in the preputial region (Table 2). *C. albicans* and *Aspergillus* spp. were isolated from the prepuce of 2 (18%) cats. The external organs of cat reproduction could be infected by three isolates concomitantly. *Scopulariopsis* could be isolated only in one case, which was accompanied by an *A. niger* isolate. The frequencies of colonies isolated from the external organs of the reproductive tract in male stray cats are shown in Table 3. It was shown that *C. krusei* had the most frequency.

**TABLE 2:** Frequency of fungal agents isolated from the external organs of male reproductive system in stray cats.

	Organs	
Fungal agents	Penis (%)	Prepuce (%)
*Candida* spp.		
*C. krusei*	1 (9)	‐
*Non‐Candida* spp.		
*Penicillum* spp.	1 (9)	3 (27)
Mixed fungal infection		
*Penicillum* spp. and *C. krusei*	1 (9)	‐
*C. parapsilosis* and *C. krusei*	1 (9)	1 (9)
*Penicillum* spp. and *Alternaria* spp.	‐	1 (9)
*C. albicans*, *Penicillum* spp. and *Aspergillus* spp.	‐	1 (9)
*C. albicans*, *Penicillum* spp., *Aspergillus* spp. and *Scopulariopsis*	‐	1 (9)
Total	4 (36)	7 (64)

**TABLE 3:** Frequencies of fungal colonies isolated from the external organs of reproductive tract in male stray cats.

Agents	Fungal colonies in male genital organs (%)		Total
	Penis	Prepuce	
***Candida* spp**.			
*C. parapsilosis*	2 (2)	6 (5)	8 (7)
*C. krusei*	26 (23)	10 (9)	36 (32)
*C. albicans*	‐	8 (7)	8 (7)
***Non‐Candida* spp**.			
*Penicillum* spp.	5 (4)	32 (29)	37 (33)
*Aspergillus* spp.	‐	12 (11)	12 (11)
*Alternaria* spp.	‐	7 (6)	7 (6)
*Scopulariopsis*	‐	4 (4)	4 (4)
Total	33 (29)	79 (71)	112 (100)

## 4 DISCUSSION

We could isolate 5 different fungal isolates from the healthy male short hair cats (Tables 2 and 3). The cats sampled could also be infected by mixed fungal agents (Table 2). However, the penis and prepuce of the healthy cats were infected by two and five different fungal isolates, respectively (Table 2). As no visible lesions were detected on the reproductive tracts of the cats in this study. It is more appropriate to say that these cats were contaminated by fungi as opposed to infection.

The results showed that 36% of the isolated microorganisms consisted of *Penicillum* spp. in single isolates (Table 2). This isolate was recovered in the genital system of female cats (Garoussi et al., 2016). This species is likely a resident member of the normal flora of the genital system in short hair cats. It was shown that fungal infection is very high in female cats (Garoussi et al., 2016). However, it is likely that the fungal infection transfers from female to male during the mating process (Azarvandi et al., 2017b).

In addition, an *A. Niger* isolate was recovered (Tables 2 and 3). Therefore, in the present study, it is suggested that this fungal infection is either transient or a natural flora of the reproductive system of cats. Nevertheless, due to the reduced number of isolates and the lack of a relationship among the different kinds of animals, the real role of the filamentous fungi in the normal male genital system flora of short hair stray cats remains to be determined in further studies. Thus, the pattern of tissue invasion seems to depend on the pathway of the fungal agent's entry and the body's defence and immunity mechanisms.

The occurrence and establishment of disease in different tissues of the body is caused by different factors. Some may be related to host factors such as age, pregnancy and species and some to environmental and climatic factors such as altitude, humidity, light, ambient temperature, weather and generally to a set of factors that are outside the functional factors, not related to the host. Moisture can be an important source for the growth of fungi. Therefore, considering that this survey was conducted in a cold and nonhumid area, the amount of isolated fungal contamination could probably be lower. Some fungal species are more frequently reported than others. This might reflect their parasitic adaptation, along with their proportionately higher abundance in the environment (Matsumoto et al., 1994). Thereafter, the tissue invasion pattern seems to depend on the fungal agent's preferred route of entry and on its subsequent ability to invade tissues (Malik et al., 2004). All these fungi are environmental saprophytes, causing infections in the presence of predisposing local or systemic factors (Matsumoto et al., 1994).

In the present study, the fungal isolates showed progressive involvement of different structures of external organs of male cats’ reproduction system, thus confirming the locally invasive nature of different isolates in agreement with previous reports in which infection did not disseminate (Garoussi et al., 2016). Therefore, it is possible that this fungal group demonstrates a tropism towards this anatomical site in cats with different ages (Table 1). Interestingly, *Penicillum* spp. and *C. Krusei* the most frequent fungal species located in penial and preputial tissues. They may have the potential to spread through the male genital system in cats. *Candida* can be localised to the mucous membranes and skin, it is distributed worldwide in different animals and is the most commonly caused by *C. albicans* and *C. Krusei* among others (Garoussi et al., 2016).

The reproduction system of different animals can be the major reservoir of yeasts such as *C. albicans* and *C. neoformans* (Chengappa et al., 1984). Fungal agents can be isolated as a secondary infection in the male genital system. However, severe fungal infections in cats are quite rare in clinical veterinary practice.

Opportunistic fungi usually require a host that is debilitated or immunosuppressed to establish infection. Prolonged administration of antimicrobials or immunosuppressive agents appears to increase the likelihood of infection by opportunistic fungi that cause disease such as aspergillosis and candidiasis, which may be focal or systemic. *C. albicans* is commonly found in balanoposthitis human patients and is responsible for up to 30.5%−35% of all cases of balanoposthitis (Hu et al., 2017).

*Alternaria* spores regularly occur in the air, which includes the ubiquitous saprobic modules. *C. parapsilosis* is not an obligate human pathogen, having been isolated from nonhuman sources such as domestic animals, insects and soil (Trofa et al., 2008). *Scopulariopsis* species are commonly found in soil, decaying wood, and various other plant and animal products (Kirk et al., 2008).

Diabetes also is a risk factor for mycosis development. In a retrospective study on urinary tract infections in 23 dogs and 12 cats, a number of medical problems were reported in the affected animals, such as diabetes and clinical signs of urinary tract infections. Seven different fungal species were isolated from diseased animals, but *C. albicans* was the most common (Jin & Lin, 2005). Some chemicals such as antibiotics and corticosteroids can cause the growth of fungus in organs. In our study, samples were taken from stray cats so there was no concurrent treatment with antibiotics or steroids, which could have predisposed to fungal infection. Diabetes can cause immunodeficiency in animals (Gostelow et al., 2014); therefore, it is possible that diabetes and immunodeficiency of any cause could be predisposing factors for genital fungal contamination such as with *C. albicans*. Hence, the question of the factors predisposing small animals to mycoses has yet to be satisfactorily answered.

In conclusion, we observed the occurrence of fungal contamination (mainly *Penicillium* and *Candida*) in the reproductive system of male cats. These microorganisms are likely to be either transient or resident in the reproductive system of stray cats. Since, male cats lack sufficient amounts of hormones that affect the immune system (such as oestrogen and progesterone). However, it is not likely the reproductive hormones play an internal role in male reproductive fungal infections in comparison to female cats. Since the unowned cats are moving in polluted and uncontrollable environments. It is possible that this contamination can be transmitted to humans if they are touched by someone. Therefore, they can be considered as an alarm in terms of public health. There are some limitations to this study including that samples were only collected from stray cats and not indoor cats or owned outdoor cats. Therefore, additional studies should be conducted on other cat populations such as indoor cats and owned outdoor cats and especially about the molecular diagnostic of the fungal species for further understanding of the role of opportunistic fungal agents in the male reproductive system of the cat. Another limitation of this study is the approximate aging system used. While we found that cats in the group estimated to be 3–4 years old had higher rates of infection compared to cats 2 years or younger or 5 years or older, this may not be accurate as age was estimated.

## AUTHOR CONTRIBUTIONS

Garoussi: research designer, conceptualisation, methodology, writing original draft, validation, supervision. Sharifzadeh: mycological culture, study execution. Khodabakhsh: sample collection, study execution. Malmasi: study execution. All authors contributed to data analysis, interpretation, the preparation of the paper and approved the final version of the manuscript.

## FUNDING

This work was supported by the Research Council of Faculty of Veterinary Medicine, University of Tehran, Tehran, Iran.

## CONFLICT OF INTEREST STATEMENT

No competing interests have been declared.

## ETHIC STATEMENTS

Permission was obtained from the Ethics Committee of Faculty of Veterinary Medicine, University of Tehran, Tehran, Iran.

### PEER REVIEW

The peer review history for this article is available at https://publons.com/publon/10.1002/vms3.1351.

## Data Availability

The data that support the findings of this research are available from the corresponding author upon reasonable request.
